# Impact of cognitive decline on medical outcomes and nursing workload: A retrospective cohort study

**DOI:** 10.1371/journal.pone.0293755

**Published:** 2023-11-22

**Authors:** Takashi Iwaanakuchi, Takuma Yoshida, Yukari Fukuda, Yumiko Uto

**Affiliations:** 1 Department of Medical Informatics, Kagoshima University Hospital, Kagoshima City, Japan; 2 Graduate School of Science and Engineering, Kagoshima University, Kagoshima City, Japan; 3 Department of Nursing, Kagoshima University Hospital, Kagoshima City, Japan; Ehime University Graduate School of Medicine, JAPAN

## Abstract

Few reports have quantitatively investigated the effect of dementia on medical outcomes and nurse workload. Therefore, we aimed to investigate whether cognitive decline can be identified from a nurse assessment and determined its effect on medical outcomes and nurse workload. This retrospective cohort study used electronic medical record data to investigate whether patients judged by nurses to have cognitive decline were as affected as those with a dementia diagnosis. Further, a model formula was created and validated to predict the probability of needing physical restraint, the nursing care workload, and the record volume. The subjects were 43,330 patients aged ≥40 years who were hospitalized at the study hospital during the four-year study period. Data were analyzed using the chi-square test, Welch’s t-test, logistic regression analysis and linear regression analysis. The implementation of physical restraint and a discharge support conference was significantly higher in patients deemed by nurses to have cognitive decline. Nurse-deemed patients with cognitive decline were affected by the outcome and workload as much as those with dementia. The false discovery rate for the Probability model formula of physical restraint and discharge support conference were 0.198 and 0.266. The correlation coefficient of the model formula for predicting nursing care and recording volume was 0.5–0.6. Combining nurse assessment and patient attribute information in a model was useful for predicting nurse workload. These findings may serve as a foundational component for the Clinical Decision Support System, aiding in the evaluation of intervention methods from the early stages of hospital admission and improving care delivery.

## Introduction

In Japan, the number of patients with dementia was estimated to be approximately 4.62 million in 2012, accounting for one in seven adults aged >65 years. Combined with the estimated 4 million people with mild cognitive impairment, approximately one in four adults aged >65 years has or is at risk of dementia [1, 2]. Demographic trends indicate that the dementia rates will continue to increase as the population ages [2]. Thus, addressing the challenges posed by dementia has become a major national concern.

Older adults with dementia present with an average of three or more complications [3]. Furthermore, owing to poor cognitive ability and judgment, patients with dementia are more prone to adverse events, such as delirium, during hospitalization and they tend to require longer hospital stays [4–7]. Additional causes of prolonged hospitalization include poorly examined discharge plans, delays due to transfer arrangements with post-discharge medical institutions, and inadequate communication and arrangements between medical staff [8, 9]. Staff involved in treatment should help resolve these problems and plan for continuing treatment and nursing from the earliest stages of hospitalization.

Prolonged hospitalization of patients with dementia in turn has been shown to increase inpatient care costs, as examined by previous studies on the financial burden of dementia [10–13]. Additionally, caring for patients with dementia, especially responding to the behavioral and psychological symptoms of dementia, presents care providers with various challenges [14–16]. For example, a previous study revealed that nurses facing these specific difficulties are prone to burnout [17]. Nevertheless, this report and similar others have solely focused on assessing the psychological strain experienced by healthcare providers, without conducting quantitative investigations into the variations in both the nature and extent of care required for patients with dementia compared to those with other conditions. Patients with dementia need to be observed carefully, as they tend to have reduced complaints and require physical restraint to protect their lives and prevent physical harm; however, the impact of caregiving responses on the maintenance of nursing records, a significant factor in determining the nursing workload, has not been disclosed. Therefore, it is important to understand the impact on record volume, especially in Japan, where workstyle reform is being promoted [18].

Previous studies have explored nursing workload within intensive care units (ICUs) across various countries using an indicator called nursing activities score (NAS) [19]. These investigations typically involved the quantitative assessment of both physical and mental workloads and clarifying their correlation, as well a comparison of findings between countries [20]. Additionally, some research has delved into the influence of various factors on workload, including nurse-related variables such as work shift and type of ICU and patient-related factors such as medical or surgical admission and referral sources [21]. However, NAS is an index designed for use in the ICU and is not appropriate for evaluating the impact of dementia-specific care on workload.

The primary obstacles to quantitatively reporting on medical outcomes (treatment results and patient outcome) and assessing the impact of dementia-specific care on nurse workload is the difficulty in identifying patients with dementia and the inability to proceed with case-control studies including patients with and without dementia. Many Japanese individuals tend to avoid psychiatric visits and cognitive function tests; even those in whom impaired cognitive function is indicated do not seek medical care in the form of tests and prescriptions. Therefore, "dementia" is rarely registered as a diagnosis name in electronic medical records, leading to a significant gap in reported diagnosis rates [22]. A Japanese study reported that the frequency of patients with a dementia diagnosis was 5.1% in electronic medical records and 1.9% in Diagnosis Procedure Combination (DPC) data, the latter of which is used as a comprehensive payment system for acute-phase hospital-admission care [23]. These frequencies are low compared to the prevalence rates obtained from epidemiological studies [1]. Therefore, it has become evident that the inability to differentiate between patients with and without dementia has caused a failure in various impact assessments due to cognitive decline.

To address this issue, nurse records offer a potential solution. Nurses in Japan recognize cognitive decline as a crucial concern and have traditionally conducted cognitive function assessments as needed during patient hospitalization [24]. Hence it is unsurprising that nurse records contain a higher percentage of cognitive function assessment data compared to records solely focused dementia care implementation [25]. In 2016, the Dementia Care Fee was newly established in the Health Care Insurance System in Japan with the aim of providing high-quality dementia care, underscoring the increasing importance of cognitive function assessment information [26].

The purpose of this study was to determine whether patients with impaired cognitive function could be identified from the assessment information of nurses at the time of admission, independent of an official diagnosis. Furthermore, we aimed to predict the impact on the nursing workload due to medical resource input and clinical outcomes by combining the presence or absence of cognitive impairment and other patient characteristics.

## Materials and methods

### Participants

The inclusion criteria for this retrospective cohort study were as follows: Patients must have had a single hospitalization at Hospital A from April 2016 to March 2020, be 40 years of age or older on the day of hospitalization, and have incurred hospitalization charges. In Japan, public nursing care insurance premiums are collected from individuals aged >40 years because of the increased risk of developing aging-related diseases at this age. Furthermore, since presenile dementia is considered an age-associated disease covered by nursing care service benefits, this survey targeted individuals aged >40 years [27]. Exclusion criteria included hospitalized patients with dental conditions and patients readmitted on the same day of discharge. Dental diseases are excluded from this study because the dental fee system differs from that of other medical diseases in Japan. Hospital A is a 653-bed government-approved special functioning hospital with the capacity to provide advanced medical care, develop advanced medical technology, and conduct training; it also functions as a tertiary referral hospital [28, 29]. Data were accessed by the investigator between May 1 and May 31, 2020.

### Measurements and data sources

Three factors were established to identify cognitive decline: Factor A, presence/absence of diagnosis name; Factor B, prescription for dementia medication, or lack thereof; and Factor C, nurse cognitive impairment assessment findings.

Factor A (diagnosis name) was defined by the International Classification of Diseases 10th Revision codes. Patients whose registration data included F00 (Alzheimer’s dementia), F01 (vascular dementia), F02 (dementia from other diseases), F03 (unspecified dementia), and G30 (Alzheimer’s disease) in the hospital disease name registration system, were regarded as having a diagnosis of dementia [30].

Factor B (prescriptions for dementia medication, or lack thereof) were assessed based on database records. This database includes medications prescribed during the hospital stay and medications bought by the patient. Drugs for treating dementia were defined in the Alzheimer’s dementia treatment (these drug codes are as follows: 1190012, 1190018, 1190019, 1190700) according to the drug code listed in the NHI price standard (code assigned to ethical drugs by the Ministry of Health, Labour, and Welfare, 2020) [31].

Factor C (nurse cognitive impairment assessment findings) was based on the "Determination of the degree of independence of activities of daily living of dementia patients" performed at admission; the presence of cognitive impairment was determined according to nurse assessment. This approach is an evaluation indicator developed by the Ministry of Health, Labour, and Welfare in 2006 [32]. This indicator is intended to allow for an objective and straightforward evaluation of dementia and independence in activities of daily living at the community level and in medical institutions by health care personnel, nurses, social, and caregiving personnel. It has been adopted as a measure of cognitive function in Japan’s nursing care insurance system [27, 33, 34]. The degree of independence in activities of daily living is assessed based on Grades I–IV and M. In this indicator, Grade I indicates that the patient has some sort of dementia, but is almost independent in daily domestic and social activities. However, in cases of Grade IV, symptoms, behavior, or communication difficulties that interfere with the patient’s daily life are observed frequently and require constant care. In addition, cases of Grade M require constant observation and specialized medical care. This analysis included patients with Grades I–IV and M who had been assessed by the nurses for cognitive impairment.

In addition to identifying cognitive impairment, six factors were established to evaluate the impact on medical outcomes and the amount invested in medical resources: degree of freedom, transit classification, age, location, disease classification, and presence or absence of surgery. The degree of freedom is a comprehensive evaluation of the patient’s activities of daily living and consists of four categories: “I: always asleep,” “II: can sit up in bed,” “III: can walk in the hospital room,” and “IV: minimal difficulties in everyday life.” Transit classification consists of three categories: “litter patient,” “escort patient,” and “independent transit patient.” A “litter patient” indicates a patient who requires support or a stretcher to move and requires two caregivers. An “escort patient” indicates a patient who needs to be accompanied or requires a wheelchair to move and requires a single caregiver. An “independent transit patient” describes patients who can move independently [35]. The location was classified according to whether a patient’s residence was within or outside the secondary medical area. In Japan, secondary medical areas are set up for completing general inpatient treatments, such as emergency medical care [36, 37]. Disease classification was performed using the Major Diagnostic Categories (MDCs), and patients were classified into 18 categories. The MDC is denoted by the first two digits of the DPC code and represents a major category of disease the patient has; for example, MDC01, MDC02, and MDC04 refer to nervous system diseases, eye disease, and respiratory system diseases, respectively. The presence or absence of surgery was determined using the DPC code [38].

### Outcome

Six items were set as the medical outcome and medical resource input: the number of days of hospital stay, physical restraints, implementation of a discharge support conference, medical costs, the type and daily frequency of nursing care provided per hospitalization, and the number of cases and characters recorded in the nursing records per day.

For patients who need support at discharge, the hospital nurses, hospital discharge coordination nurses, and medical social workers, among others, jointly hold a discharge support conference during the patient’s hospital stay to address patient problems and anticipated place of discharge and to discuss the method for providing necessary medical/caregiving services after discharge. The implementation of the Discharge Support Conference was set as one of the outcomes. Medical costs include the cost of medical materials and medications used for care.

Kagoshima University Hospital has created a list of nursing care categories and items for electronic medical records based on the nursing work categories created by the Japanese Nursing Association. Care items are divided into three levels: large, medium, and small. The small category includes assistance with activities of daily living, such as cleaning and eating assistance; medical assistance, such as drainage management; blood draws; and observation items, such as temperature and nausea [35, 39–41]. When calculating the type and daily frequency of nursing care provided per hospitalization, we aggregated the number of small category items of nursing care provided. The number of nurse-recorded characters was calculated by a byte count. English characters and numbers were regarded as 1 byte, whereas Japanese hiragana and kanji characters were regarded as 2 bytes.

### Ethical considerations

This study was conducted in accordance with the tenets of the Declaration of Helsinki. This study was approved by the Ethics Committee on Epidemiological Studies of Kagoshima University (Approval Number: 190154). During collection of information, any data that could be used to identify individuals were excluded, and data analysis was conducted treating the data as anonymous processing information. The need for patient consent was waived owing to the retrospective study design and data anonymization. Reporting of this study was guided by the Strengthening the Reporting of Observational Studies in Epidemiology Statement [42].

### Statistical analyses

The status of patients with Factors A–C was confirmed. Those with factors A-C were considered exposure groups, and those without factors A-C were considered non-exposure groups, and comparisons were made based on the presence or absence of each factor. The effect of the two-value dependent variable of physical restraint and implementation of discharge support conferences was confirmed by classifying patients with and without surgery and performing the chi-square test according to the presence or absence of Factors A–C. When the dependent variables were hospitalization duration (days), medical care costs, type and frequency of nursing care, number of cases, and number of characters in the nursing records, the participants were categorized by disease and surgery. Welch’s t-test was performed according to the presence or absence of Factors A–C. The disease state was classified using the first six digits of the DPC code, which corresponds to the medium classification of the disease name. Outlier data at the 95th percentile quartile were excluded. The level of significance was set at p<0.05.

Explanatory variables other than age (nominal and ordinal scales) were quantified and modeled using variables (Table 1). For the two-value dependent variables (physical restraint and discharge support conference), the number of cases in the no-implementation group was considerably larger than that in the yes-implementation group, resulting in imbalanced data. Therefore, an appropriate regression model could not be created. Thus, the number of cases in the no-implementation group was randomly reduced to the same number of cases in the implementation group, and logistic regression analysis was conducted. Linear regression analysis was performed for the other continuous dependent variables. The estimated weight factors for the 29 items of explanatory variables were determined, and the probability model formula to predict the implementation of physical restraint and discharge support conference was determined. In addition, a model formula to predict the number of days of hospital stay, medical costs, type and frequency of nursing care, number of cases, and number of characters in nursing records was determined and validated. The model formula for the two-value dependent variable used the false discovery rate (FDR), positive predictive value (PPV), negative predictive value (NPV), sensitivity, specificity, and five-fold cross-validation. In contrast, the model formula for the continuous dependent variable used the square root of the coefficient of determination *R*^2^ (the criterion corresponding to the correlation coefficient) and five-fold cross-validation.

**Table 1:** Explanatory variables used in the model formula.

Diagnosis name of dementia		
	X1	1 if diagnosed, 0 if not
Treatments		
	X2	1 with medication, 0 without
Assessment by nurse		
	X3	1 if assessed to have cognitive impairment, 0 if not
Degree of freedom (I as criterion)		
	X41	1 if II, 0 if other
	X42	1 if III, 0 if other
	X43	1 if IV, 0 if other
Transit classification (litter patient as criterion)		
	X51	1 if escort, 0 if other
	X52	1 if independent, 0 if other
Age		
	X6	
Medical area		
	X7	1 if within secondary medical area, 0 if outside
Disease classification (MDC01 of DPC as criterion)		
	X802	1 if MDC02, 0 if other
	X803	1 if MDC03, 0 if other
	X804	1 if MDC04, 0 if other
	X805	1 if MDC05, 0 if other
	X806	1 if MDC06, 0 if other
	X807	1 if MDC07, 0 if other
	X808	1 if MDC08, 0 if other
	X809	1 if MDC09, 0 if other
	X810	1 if MDC10, 0 if other
	X811	1 if MDC11, 0 if other
	X812	1 if MDC12, 0 if other
	X813	1 if MDC13, 0 if other
	X814	1 if MDC14, 0 if other
	X815	1 if MDC15, 0 if other
	X816	1 if MDC16, 0 if other
	X817	1 if MDC17, 0 if other
	X818	1 if MDC18, 0 if other
Presence or absence of surgery		
	X9	1 if underwent surgery, 0 if no surgery

Abbreviations: DPC, Diagnosis Procedure Combination; MDC, Major Diagnostic Categories

All data analyses were performed using R software version 3.6.0 (R Software for Statistical Computing, Vienna, Austria) [43].

## Results

We extracted the data of 43,330 participants from the hospital information database. The number of individuals corresponding to each explanatory variable is shown in Table 2. The discharge support conference was implemented for 7,816 patients while physical restraint was implemented for 6,382 patients. The average hospitalization duration was 14.6 days, and the average cost of medical treatment per hospital admission was 366,396 yen. On average, 37.0 types of nursing care were delivered per hospital admission, 87.3 times a day. On average, nursing records were recorded 8.9 times a day, which amounted to approximately 402.5 bytes.

**Table 2:** Results of simple aggregation of the explanatory variable and dependent variables.

	n	(%)	average	(SD)
Factors and covariates				
With diagnosis name of dementia	770	(1.8%)		
With dementia treatment	642	(1.5%)		
With assessment of cognitive impairment by nurse	1,441	(3.3%)		
Degree of freedom				
I (bedridden)	2,891	(6.7%)		
II (can sit up in bed)	5,155	(11.9%)		
III (can walk around the hospital room)	15,629	(36.1%)		
IV (almost no disability in daily life)	19,199	(44.3%)		
Transit classification				
1_Litter	4,150	(9.6%)		
2_Escort	14,495	(33.5%)		
3_Independent	24,237	(55.9%)		
Average age (SD)			66.2	(11.9)
Living in secondary medical area	20,303	(46.9%)		
Disease classification				
MDC01	2,838	(6.5%)		
MDC02	4,388	(10.1%)		
MDC03	1,835	(4.2%)		
MDC04	4,742	(10.9%)		
MDC05	4,225	(9.8%)		
MDC06	9,401	(21.7%)		
MDC07	3,046	(7.0%)		
MDC08	961	(2.2%)		
MDC09	584	(1.3%)		
MDC10	1,831	(4.2%)		
MDC11	2,099	(4.8%)		
MDC12	3,341	(7.7%)		
MDC13	1,376	(3.2%)		
MDC14	109	(0.3%)		
MDC15	3	(0.0%)		
MDC16	862	(2.0%)		
MDC17	410	(0.9%)		
MDC18	744	(1.7%)		
With surgery	20,801	(48.0%)		
Outcome				
Implementation of discharge support conference	7,816	(18.0%)		
Implementation of physical restraint	6,382	(14.7%)		
Average number of days of hospital stay (SD)			14.6	(17.1)
Average medical cost per hospital admission (SD)			366,396	(722,052)
Average number of types of nursing care per hospital admission (SD)			37.0	(15.4)
Average frequency of nursing care provided per day (SD)			87.3	(47.4)
Average number of cases in nursing records per day (SD)			8.9	(4.4)
Average number of characters by bytes in nursing records per day (SD)			402.5	(222)

Abbreviations: MDC, Major Diagnostic Categories; SD, Standard deviation

* For n, the percentage of eligible patients is also listed. For the average, the standard deviation (SD) is also listed.

### Factors indicative of cognitive impairment

Among the factors that identified cognitive impairment, 770 participants had Factor A, 642 participants had Factor B, and 1,441 participants had Factor C. In total, 1,851 patients had at least one of the factors, while 263 patients had all factors.

### Impact of Factors A, B, and C on physical restraints and discharge support

Depending on the presence or absence of Factors A–C, we verified differences in the implementation rates of physical restraint and discharge support conference by the presence of surgery. Comparing the presence or absence of Factors A–C, and history of surgery, the rates of physical restraint and discharge support conferences were significantly higher for patients with than for those without these factors (Table 3). For example, the implementation rates of physical restraint in the group without surgery were 28.3% in patients with Factor A and 7.4% in those without, 32.6% in patients with Factor B and 7.5% in those without, and 40.4% in patients with Factor C and 6.7% in those without.

**Table 3:** Rates of physical restraint and discharge support conference by whether or not a factor of cognitive impairment.

			With factor		Without factor		p-value
Factor A: diagnosis name of dementia							
	No surgery		n = 495		n = 21,499		
		With physical restraint	140	(28.3%)	1,596	(7.4%)	<0.001
		With conference	274	(55.4%)	3,134	(14.6%)	<0.001
	With surgery		n = 273		n = 20,528		
		With physical restraint	159	(58.2%)	4,379	(21.3%)	<0.001
		With conference	142	(52.0%)	4,130	(20.1%)	<0.001
Factor B: dementia treatment							
	No surgery		n = 356		n = 21,638		
		With physical restraint	116	(32.6%)	1,620	(7.5%)	<0.001
		With conference	201	(56.5%)	3,207	(14.8%)	<0.001
	With surgery		n = 283		n = 20,518		
		With physical restraint	145	(51.2%)	4,393	(21.4%)	<0.001
		With conference	163	(57.6%)	4,109	(20.0%)	<0.001
Factor C: assessment of cognitive impairment by nurse							
	No surgery		n = 778		n = 21,216		
		With physical restraint	314	(40.4%)	1,422	(6.7%)	<0.001
		With conference	461	(59.3%)	2,947	(13.9%)	<0.001
	With surgery		n = 658		n = 20,143		
		With physical restraint	356	(54.1%)	4,182	(20.8%)	<0.001
		With conference	396	(60.2%)	3,876	(19.2%)	<0.001

Abbreviations: MDC, Major Diagnostic Categories; SD, Standard deviation

### Impact of Factors A, B, and C on hospital stay, costs, and nursing care and records

The average hospital stay was 15.8 days without Factor A (diagnosis name) and 18.1 days with Factor A; 15.3 days without Factor B (treatments) and 15.6 days with Factor B; 15.3 days without Factor C (cognitive impairment by nurse assessment) and 16.7 days with Factor C. Factor A significantly impacted the length of hospital stay, but Factors B and C did not.

The average medical costs were 306,015 yen without Factor A (diagnosis name) and 290,626 yen with Factor A; 285,517 yen without Factor B (treatments) and 238,622 yen with Factor B; 292,102 yen without Factor C (cognitive impairment by nurse assessment) and 264,739 yen with Factor C. These differences were not statistically significant.

**Fig 1:**
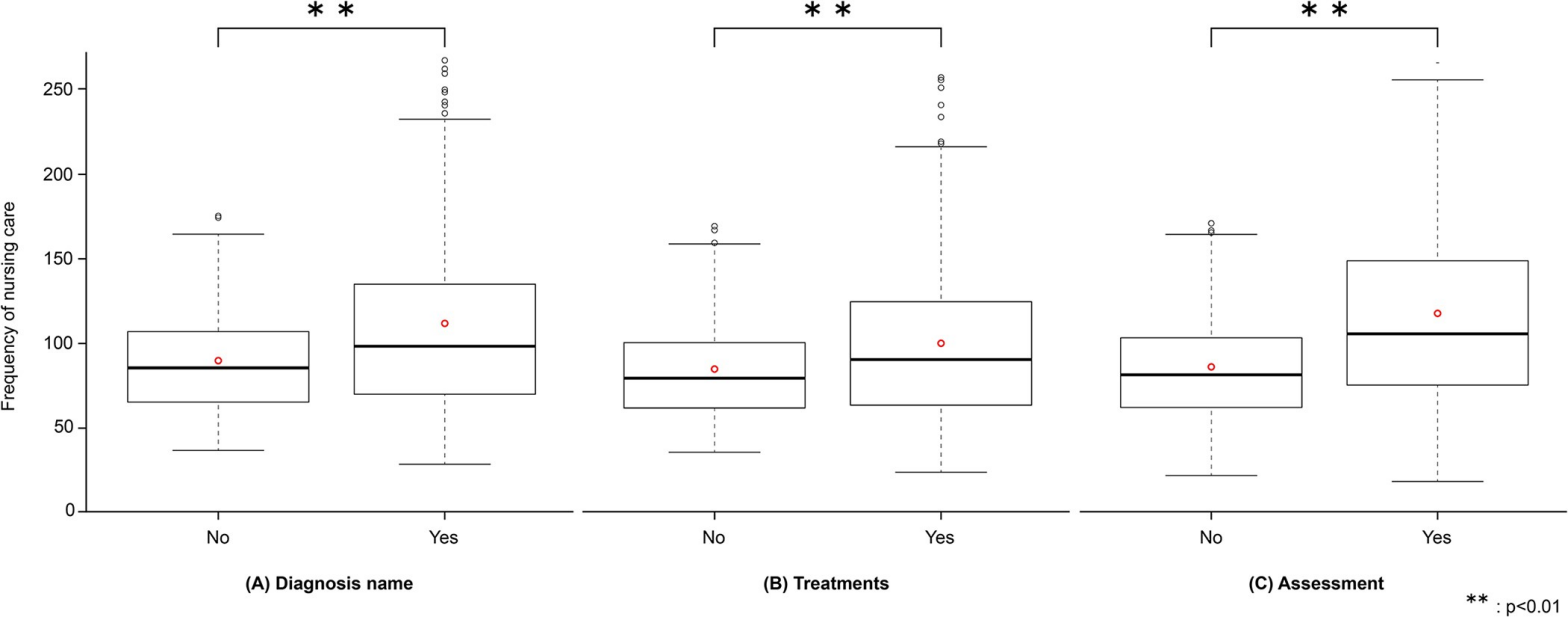
Frequency of nursing care provided per day according to whether or not the patients have factors indicative of impaired cognitive function.

The number of nursing care types provided per hospital admission was 39.0 types without Factor A (diagnosis name) vs. 45.7 with Factor A, 38.2 types without Factor B vs. 42.8 types with Factor B, and 38.5 types without Factor C vs. 46.1 types with Factor C. The frequency of nursing care provided per day was 89.6 times without Factor A vs. 111.9 times with Factor A, 84.6 times without Factor B vs. 99.9 times with Factor B, and 86.2 times without Factor C vs. 118.1 times with Factor C (Fig 1). For all factors, the number of types and frequency of nursing care provided were greater for patients with than for those without those factors. Particularly, the number of patients deemed to have cognitive impairment by nurse assessment tended to have the highest type and frequency of nursing care provided.

The number of cases in the nursing records per day was 9.1 cases without Factor A and 12.4 cases with Factor A; 8.8 cases without Factor B and 11.3 cases with Factor B; and 8.8 cases without Factor C (cognitive impairment by nurse assessment) and 12.6 cases with Factor C. The number of characters in nursing records per day was 413 bytes without Factor A vs. 588 bytes with Factor A; 396 bytes without Factor B vs. 530 bytes with Factor B; and 398 bytes without Factor C vs. 595 bytes with Factor C. For all factors, the number of cases and characters in nursing records was significantly higher for patients with factors than for those without. In particular, patients deemed as having cognitive impairment by nurse assessment had the highest case and character counts in the nursing records.

### Logistic regression analysis for physical restraints and discharge support

Logistic regression analysis was conducted to confirm the impact of the factors leading to the implementation of physical restraint and a discharge support conference. For the dependent variable “physical restraint,” the estimates for each factor identifying cognitive impairment were as follows: Factor A: 0.826 (*p*<0.001), Factor B: 0.236 (n.s.), and Factor C: 1.407 (*p*<0.001); Factor C had the strongest effect (Table 4). Other factors with significant impact were surgery (2.583, *p*<0.001), MDC02 as disease classification (-4.829, *p*<0.001), MDC09 (-3.203, *p*<0.001), and MDC08 (-3.120, *p*<0.001). In the results of the discharge support conference analysis, the estimates for each factor identifying cognitive impairment were as follows: Factor A: 0.632 (*p*<0.001); Factor B: 0.881 (*p*<0.001); and Factor C: 0.871 (*p*<0.001) (S1 Table).

**Table 4:** Results of regression analysis using physical restraint as a dependent variable.

Explanatory variable	Parameter	Estimate	Std. Error	z value	Pr (>|t|)	
(Intercept)	β0	1.72041	0.19646	8.75684	2.01E-18	***
A: Dementia disease name	β1	0.82612	0.23343	3.53901	0.00040	***
B: Dementia treatment	β2	0.23576	0.25393	0.92847	0.35316	
C: Assessment by nurse	β3	1.40704	0.16227	8.67084	4.29E-18	***
Degree of freedom II	β41	-0.68862	0.14785	-4.65771	0.00000	***
Degree of freedom III	β42	-1.61930	0.16236	-9.97346	1.99E-23	***
Degree of freedom IV	β43	-1.96997	0.16886	-11.66650	1.89E-31	***
Transit classification Escort	β51	-1.67929	0.13254	-12.66965	8.71E-37	***
Transit classification Independent	β52	-2.06040	0.14565	-14.14661	1.96E-45	***
Age	β6	0.02276	0.00230	9.90140	4.1E-23	***
Living in secondary medical area	β7	0.05819	0.05045	1.15351	2.49E-01	
MDC02	β802	-4.82891	0.15432	-31.29167	6.06E-215	***
MDC03	β803	-2.18004	0.15452	-14.10820	3.38E-45	***
MDC04	β804	-1.03122	0.11197	-9.20974	3.27E-20	***
MDC05	β805	-1.43044	0.11320	-12.63584	1.34E-36	***
MDC06	β806	-2.33871	0.10774	-21.70599	1.8E-104	***
MDC07	β807	-2.12508	0.12617	-16.84339	1.17E-63	***
MDC08	β808	-3.12013	0.22839	-13.66137	1.73E-42	***
MDC09	β809	-3.20347	0.25320	-12.65203	1.09E-36	***
MDC10	β810	-1.32656	0.14876	-8.91729	4.78E-19	***
MDC11	β811	-2.57172	0.15792	-16.28542	1.25E-59	***
MDC12	β812	-3.07486	0.15730	-19.54762	4.32E-85	***
MDC13	β813	-2.37987	0.17506	-13.59474	4.3E-42	***
MDC14	β814	-0.84336	0.40929	-2.06052	0.03935	*
MDC15	β815	8.38705	177.82852	0.04716	0.96238	
MDC16	β816	-1.53636	0.18845	-8.15274	3.56E-16	***
MDC17	β817	-0.06512	0.24198	-0.26911	0.78784	
MDC18	β818	-1.32099	0.20501	-6.44347	1.17E-10	***
With surgery	β9	2.58309	0.06424	40.20963	0	***

*: p<0.05

**: p<0.01

***: p<0.001

Abbreviations: MDC, Major Diagnostic Categories

### Linear regression analysis for hospital stay, costs, and nursing care and records

In the results of the linear regression analysis of hospitalization duration, Factor A had a statistically significant effect, with an estimated value of 5.119. Among the dependent variables, regression analysis showed that Factor C was statistically significant for all variables except for hospitalization duration (S2 Table).

The estimates for each factor that identifies cognitive impairment in the analysis of medical costs were Factor A: 97,769 (*p*<0.001); Factor B: -78,084 (*p*<0.05); and Factor C: -93,098 (*p*<0.001). Other important factors and their significant impact were estimated to be 349,099 (*p*<0.001) for surgery, -698,324 (*p*<0.001) for MDC02 as disease classification, -517,055 (*p*<0.001) for MDC09, and -433,938 (*p*<0.001) for MDC06 (S3 Table).

In the frequency analysis of nursing care provided per day, the estimates for each factor identifying cognitive impairment were as follows: Factor A: 8.66 (*p*<0.001); Factor B: -4.74 (*p*<0.05); and Factor C: 13.97 (*p*<0.001) (S4 Table). Similar results were obtained for the types of nursing care provided per hospital admission.

The analysis of the number of characters in nursing records per day showed statistically significant results for Factor A (estimate of 83.09) and Factor C (estimate of 112.79). Similar results were obtained for the number of cases of nursing records per day (S5 Table).

### Probability model formula for physical restraints and discharge support

Based on the estimated weight factor of the 29-item explanatory variables, the probability model formula that predicts implementation of physical restraint and discharge support conference was determined (Formula 1).


$$P(Y=1|x_{1},x_{2},\ldots ,x_{9})=\frac{exp[\beta_{0}+\beta_{1}x_{1}+\cdots +\beta_{9}x_{9}]}{1+exp[\beta_{0}+\beta_{1}x_{1}+\cdots +\beta_{9}x_{9}]}$$ Formula 1

The validity of the model formula was evaluated by dividing the dataset into training and validation sets. Then, the probability model formula was constructed with data for model estimation. The probability was calculated using the verification data; if the probability was >0.5, then, Y was 1; if the probability was <0.5, then, Y was 0. Based on the number of incidences of physical restraint and implementation of discharge support conferences and the results of a model-based prediction, the number of false positives and false negatives was determined. The overall FDR was calculated using Formula 2.


$$FDR=\frac{FPN+FNN}{n^{*}}$$ Formula 2

In addition, the PPV, NPV, sensitivity, and specificity values were calculated. The calculation results using five-fold cross-validation showed that the FDR, PPV, NPV, sensitivity, and specificity were 0.198, 0.795, 0.810, 0.815, and 0.790 for physical restraint, respectively. For discharge support conferences, the scores were 0.266, 0.721, 0.749, 0.764, and 0.704. The Monte Carlo simulation was repeated 1,000 times, and the standard error (SE) for each value was <0.025.

### Prediction model formula for hospital stay, costs, and nursing care and records

The prediction model formula when the dependent variable is the actual value is Formula 3.


$$E(Y|x_{1},x_{2},\ldots ,x_{9})=\beta_{0}+\beta_{1}x_{1}+\cdots +\beta_{9}x_{9}$$ Formula 3

As in the previous section, the data set was divided into testing and validation sets for validation purposes. After calculating the predicted value with the model formula, the coefficient of determination *R*^2^ was calculated, as seen in Formula 4.


$$R^{2}=1-\frac{\sum_{i=1}^{n^{*}}{\left(Y_{i}^{*}-{\bar{Y}}_{i}^{*}\right)}^{2}}{\sum_{i=1}^{n^{*}}{\left(Y_{i}^{*}-{\bar{Y}}^{*}\right)}^{2}}$$ Formula 4

The coefficient of determination *R*^2^ is a value ranging from 0 to 1. In this validation, the square root $(\sqrt{R^{2})}$ of the coefficient of determination *R*^2^, which is equivalent to the correlation coefficient, was determined. The correlation coefficients calculated by five-fold cross-validation were 0.538 (SE: 0.009) for the number of types of nursing care provided per hospital admission, 0.590 (SE: 0.007) for the frequency of nursing care provided per day, 0.553 (SE: 0.007) for the number of cases of nursing records entry per day, and 0.550 (SE: 0.010) for the number of characters of nursing records entry per day. Conversely, the coefficient was 0.404 (SE: 0.036) for the number of days in the hospital and 0.440 (SE: 0.013) for medical costs. Prediction accuracy for outcomes such as the number of days in the hospital and medical costs was low compared to workload predictions, such as nursing care and nursing records.

## Discussion

Despite the presence of cognitive decline, dementia is less likely to be registered in electronic medical records as the diagnosis owing to its low treatment priority. Therefore, this study was initiated with the hypothesis that nurse assessment information could be used to identify patients with cognitive impairment. Our results showed that the nurse assessment information could be used to identify a greater number of patients with cognitive impairment compared to assessments based on diagnoses or dementia medications. In addition, the comparison of medical outcomes and resources invested, according to the presence or absence of factors, showed significant differences in all dependent variables other than hospital stay duration and medical costs. The degree of the difference was greater than or equal to that of when diagnosis names or treatments were compared as factors. In Japan, there is currently no comprehensive quantitative indicator for measuring nurse workload. However, when we used the number of care interventions provided and the volume of nursing records as workload indicators, we observed that these indicators tend to rise for patients with decreased cognitive function.

The lack of a significant difference in medical costs, with and without nurse assessment, and the negative estimates resulting from regression analysis when medical costs were used as a dependent variable, can be attributed to several factors. Patients with cognitive impairment often struggle to communicate concerning symptoms, such as pain. This issue may delay the discovery of other diseases related to pain and reduce the provision of symptomatic treatments; therefore, it is a major limitation that impacts medical costs, as necessary tests and treatments cannot be performed as patients with cognitive impairment struggle to follow the instructions from medical staff [44]. Prior reports have indicated that the more severe the cognitive impairment, the fewer people report their pain, resulting in a lower provision of medication to relieve pain. Other reports have indicated that patients with dementia used medical services less frequently than people without cognitive issues despite having a more serious degree of physical disease [45, 46]. The results of this analysis reaffirm that a decline in cognitive function does not lead to an increase in the use of medical materials and pharmaceuticals.

In Japan, there is a tendency for dementia diagnoses to be unregistered, and several reports have suggested that there are insufficient standardized data to identify patients with dementia in other countries as well [22, 47]. Even in the United Kingdom, which has a national strategy to combat dementia, only approximately one-third to one-half of patients with dementia have been officially diagnosed [13]. Patients are generally not admitted to the hospital for dementia treatment, and dementia is not a priority for treatment or care [48]. Conversely, nurses have traditionally considered patient cognitive function as vital patient information. The assessment of whether the patient’s level of activities of daily living is independent, whether the patient can manage their medications by themselves, and whether there is a risk of falls or tube removal is carried out at the early stages after hospital admission, and a nursing plan is formulated thereafter. Many countries are concerned regarding the increases in dementia rates because of the increased burden on care providers, which is consistent with the patient attributes that nurses assess at the time of admission; these assessments may help increase the future rates of accurate patient classification.

The validation of the probability model formula for physical restraint and discharge support conferences constructed in this study confirmed a certain level of accuracy. Currently, in Japan, all hospitalized patients are assessed for the necessity of physical restraint and discharge support conferences. While there is a need to improve work efficiency, we believe that this model formula could be used to determine the priority of patients who should be considered for early intervention in discharge support and alternative measures other than physical restraint for the sake of medical safety.

In assessing the validity of the prediction model formula where the dependent variable is a continuous parameter, the correlation coefficients for nursing care workload and nursing record volume were >0.5. This result indicates a correlation, thus, confirming the validity of the model to a certain extent. Conversely, regarding the hospitalization duration and medical costs, the correlation coefficients were 0.4–0.5. This suggests a moderate degree of influence for each explanatory variable used in this analysis. Further improvement in accuracy can be anticipated by fine-tuning or adding additional variables to the model.

Among the factors indicative of cognitive function, we evaluated the validity of the model formula by excluding the nurse assessment data and found the accuracy of both dependent variables to be reduced. Thus, the assessment information of nurses is an important factor in estimating medical outcomes and the number of medical resources to be invested.

In Japan, we use a large-scale database with high universality regarding health and medical care, such as medical insurance premium billing data and DPC data, which are used to design systems in the field of insurance medicine. Conversely, the assessment information (explanatory variables) of nurses used in this study and the number of human resources (dependent variables) are not included in these databases, and there are insufficient data to enable policymakers to understand the actual situation of the patients. The results of this study are useful, as they quantitatively demonstrate the impact of patients suffering from dementia on care providers.

In addition, the explanatory variables used in this analysis include data that many medical institutions can obtain on the 1st day of admission. The model formula obtained in this study is thus expected to be used as an engine for the Clinical Decision Support System to examine intervention methods from the early stages of hospital admission and in the system for providing care.

This study has several limitations. First, the data analyzed are limited to a single hospital. However, in this study, each patient has a DPC code indicative of the patient’s attribute information. The case mix classification method, DPC, is a system applied to 85% of acute care hospital beds in Japan. As the classification method is strictly standardized, the results of this study will aid in estimating the outcomes of patients in other hospitals with the same DPC codes. Second, the appropriateness of the outcomes set is another limitation. In Japan, there are no standard objective indicators to determine the workload and sense of burden of nurses. In this study, indicators, such as nursing care implementation data, text volume, and physical restraints, were set based on previous studies and the results of a survey conducted by the Ministry of Health, Labour, and Welfare [14–18]. However, caution must be exercised in applying these results to all acute hospitals. Finally, although the validity evaluation of the model formula obtained in this study achieved a certain degree of accuracy, the predictive value of the model formula alone is not sufficient to determine the provision of care and staffing to patients. We plan to verify the model formula by selecting more appropriate independent variables from the data that exist in the hospital information system on the day of admission and adjusting for multicollinearity and confounding factors.

## Conclusions

In this study, it was revealed that nurse assessment information could identify patients with cognitive impairment without the need for an official diagnosis. The definition of cognitive impairment by nurses was found to have an impact on medical outcomes and medical resource usage to an extent equal to that of an official dementia diagnosis. These results also indicate that incorporating the nurses’ assessment information and patient characteristics into a model formula could be useful in estimating the amount of nursing work required to manage individual patients. Even within the Japanese healthcare system, where the registration of dementia and unstable emotional states in medical records is challenging, it is possible to identify this patient population from nurse record data. If patient classification is performed using nursing information, as in this study, analysis using real-world data can be further promoted. This, in turn, will be beneficial when considering staffing decisions for nursing providers and devising intervention methods tailored for this specific population.

## Supporting information

S1 Table Results of regression analysis using discharge support conferences as a dependent variable. (DOCX)

S2 Table Results of regression analysis using the hospital stay length as a dependent variable. (DOCX)

S3 Table Results of regression analysis using the medical costs as a dependent variable. (DOCX)

S4 Table Results of regression analysis using the frequency of nursing care provided per day as a dependent variable. (DOCX)

S5 Table Results of regression analysis using the number of characters in nursing records per day as a dependent variable. (DOCX)

S1 File Raw data of explanatory and dependent variables of participants. (XLSX)
