# Supplementary material for: Impact of cognitive decline on medical outcomes and nursing workload: A retrospective cohort study
Source: PLoS One. 2023 Nov 22;18(11):e0293755. doi: 10.1371/journal.pone.0293755 (PMC10664958; doi:10.1371/journal.pone.0293755)
Supplement: S1 Table — (DOCX) [file pone.0293755.s001.docx]

S1 Table. Results of Regression Analysis Using Discharge Support Conferences as a Dependent Variable.

| Explanatory variable | Para-  meter | Estimate | Standard error | z value | Pr (>\|t\|) |  |
| --- | --- | --- | --- | --- | --- | --- |
| (Intercept) | β0 | -0.68780 | 0.15009 | -4.58260 | 4.59E-06 | *** |
| A: Dementia disease name | β1 | 0.63231 | 0.18951 | 3.33647 | 0.00085 | *** |
| B: Dementia treatment | β2 | 0.88087 | 0.22111 | 3.98384 | 0.00007 | *** |
| C: Assessment by nurse | β3 | 0.87124 | 0.12504 | 6.96742 | 3.23E-12 | *** |
| Degree of freedom II | β41 | 0.02953 | 0.11220 | 0.26318 | 0.79241 |  |
| Degree of freedom III | β42 | -0.71443 | 0.12117 | -5.89616 | 3.72E-09 | *** |
| Degree of freedom IV | β43 | -1.42562 | 0.12692 | -11.23264 | 2.82E-29 | *** |
| Transit classification Escort | β51 | -0.47200 | 0.10219 | -4.61881 | 0.00000 | *** |
| Transit classification Independent | β52 | -1.29527 | 0.11218 | -11.54664 | 7.68E-31 | *** |
| Age | β6 | 0.03989 | 0.00182 | 21.89222 | 3.08E-106 | *** |
| Living in secondary medical area | β7 | -0.03250 | 0.04010 | -0.81038 | 0.41772 |  |
| MDC02 | β802 | -2.40339 | 0.10640 | -22.58738 | 5.77E-113 | *** |
| MDC03 | β803 | -1.20310 | 0.12605 | -9.54478 | 1.36E-21 | *** |
| MDC04 | β804 | -0.90960 | 0.09103 | -9.99182 | 1.66E-23 | *** |
| MDC05 | β805 | -1.22894 | 0.09356 | -13.13595 | 2.05E-39 | *** |
| MDC06 | β806 | -1.15436 | 0.08447 | -13.66538 | 1.63E-42 | *** |
| MDC07 | β807 | -0.44472 | 0.09697 | -4.58628 | 0.00000 | *** |
| MDC08 | β808 | -0.64071 | 0.15027 | -4.26360 | 0.00002 | *** |
| MDC09 | β809 | -1.32234 | 0.19016 | -6.95387 | 3.55E-12 | *** |
| MDC10 | β810 | -0.57298 | 0.12267 | -4.67107 | 3.00E-06 | *** |
| MDC11 | β811 | -0.90559 | 0.11573 | -7.82504 | 5.07E-15 | *** |
| MDC12 | β812 | -1.18189 | 0.11086 | -10.66070 | 1.55E-26 | *** |
| MDC13 | β813 | -0.44705 | 0.12315 | -3.63005 | 0.00028 | *** |
| MDC14 | β814 | -0.95792 | 0.43614 | -2.19639 | 0.02806 | * |
| MDC15 | β815 | -12.47025 | 119.46808 | -0.10438 | 0.91687 |  |
| MDC16 | β816 | -0.28886 | 0.15145 | -1.90735 | 0.05648 |  |
| MDC17 | β817 | 1.93826 | 0.20820 | 9.30982 | 1.28E-20 | *** |
| MDC18 | β818 | -0.54479 | 0.16748 | -3.25297 | 0.00114 | ** |
| With surgery | β9 | 0.75363 | 0.04359 | 17.28851 | 5.74E-67 | *** |
| *: p<0.05　　**: p<0.01　　***: p<0.001 | | | | | | |

Abbreviations: MDC, Major Diagnostic Categories
