# Supplementary material for: Impact of cognitive decline on medical outcomes and nursing workload: A retrospective cohort study
Source: PLoS One. 2023 Nov 22;18(11):e0293755. doi: 10.1371/journal.pone.0293755 (PMC10664958; doi:10.1371/journal.pone.0293755)
Supplement: S2 Table — (DOCX) [file pone.0293755.s002.docx]

S2 Table. Results of Regression Analysis Using the Hospital Stay Length as a Dependent Variable.

| Explanatory variable | Parameter | Estimate | Standard error | t value | Pr (>\|t\|) |  |
| --- | --- | --- | --- | --- | --- | --- |
| (Intercept) | β0 | 24.99376 | 0.60169 | 41.53900 | <2e-16 | *** |
| A: Dementia disease name | β1 | 5.11861 | 0.71614 | 7.14700 | 8.98E-13 | *** |
| B: Dementia treatment | β2 | -0.58541 | 0.76959 | -0.76100 | 0.44685 |  |
| C: Assessment by nurse | β3 | 0.82088 | 0.48476 | 1.69300 | 9.04E-02 | . |
| Degree of freedom II | β41 | -1.32210 | 0.45445 | -2.90900 | 3.63E-03 | ** |
| Degree of freedom III | β42 | -2.65220 | 0.49552 | -5.35200 | 8.73E-08 | *** |
| Degree of freedom IV | β43 | -3.82251 | 0.51356 | -7.44300 | 1E-13 | *** |
| Transit classification Escort | β51 | -2.42129 | 0.41567 | -5.82500 | 5.75E-09 | *** |
| Transit classification Independent | β52 | -3.46915 | 0.45186 | -7.67800 | 1.66E-14 | *** |
| Age | β6 | 0.00939 | 0.00681 | 1.38100 | 1.67E-01 |  |
| Living in secondary medical area | β7 | -1.00982 | 0.15130 | -6.67400 | 2.51E-11 | *** |
| MDC02 | β802 | -16.76448 | 0.39514 | -42.42700 | <2e-16 | *** |
| MDC03 | β803 | -4.58726 | 0.46836 | -9.79400 | <2e-16 | *** |
| MDC04 | β804 | -6.38013 | 0.37309 | -17.10100 | <2e-16 | *** |
| MDC05 | β805 | -10.50841 | 0.38358 | -27.39600 | <2e-16 | *** |
| MDC06 | β806 | -8.92772 | 0.34216 | -26.09200 | <2e-16 | *** |
| MDC07 | β807 | -4.66272 | 0.40805 | -11.42700 | <2e-16 | *** |
| MDC08 | β808 | -8.93272 | 0.58001 | -15.40100 | <2e-16 | *** |
| MDC09 | β809 | -13.86205 | 0.71815 | -19.30200 | <2e-16 | *** |
| MDC10 | β810 | -8.60481 | 0.46938 | -18.33200 | <2e-16 | *** |
| MDC11 | β811 | -8.94035 | 0.45276 | -19.74600 | <2e-16 | *** |
| MDC12 | β812 | -12.19646 | 0.40414 | -30.17900 | <2e-16 | *** |
| MDC13 | β813 | 1.97532 | 0.51169 | 3.86000 | 0.00011 | *** |
| MDC14 | β814 | -8.90650 | 1.50530 | -5.91700 | 3.31E-09 | *** |
| MDC15 | β815 | -20.00653 | 8.85108 | -2.26000 | 2.38E-02 | * |
| MDC16 | β816 | -9.81281 | 0.60541 | -16.20900 | <2e-16 | *** |
| MDC17 | β817 | 37.56952 | 0.83024 | 45.25200 | <2e-16 | *** |
| MDC18 | β818 | -5.82518 | 0.64925 | -8.97200 | <2e-16 | *** |
| With surgery | β9 | 5.92683 | 0.16329 | 36.29700 | <2e-16 | *** |

*: p<0.05, **: p<0.01, ***: p<0.001

MDC, Major Diagnostic Categories
