# Supplementary material for: Impact of cognitive decline on medical outcomes and nursing workload: A retrospective cohort study
Source: PLoS One. 2023 Nov 22;18(11):e0293755. doi: 10.1371/journal.pone.0293755 (PMC10664958; doi:10.1371/journal.pone.0293755)
Supplement: S3 Table — (DOCX) [file pone.0293755.s003.docx]

S3 Table. Results of Regression Analysis Using the Medical Costs as a Dependent Variable.

| Explanatory variable | Parameter | Estimate | Standard error | t value | Pr (>\|t\|) |  |
| --- | --- | --- | --- | --- | --- | --- |
| (Intercept) | β0 | 767,003 | 24,505 | 31.300 | <2e-16 | *** |
| A: Dementia disease name | β1 | 97,769 | 29,166 | 3.352 | 0.00080 | *** |
| B: Dementia treatment | β2 | -78,084 | 31,343 | -2.491 | 0.01273 | * |
| C: Assessment by nurse | β3 | -93,098 | 19,743 | -4.716 | 2.42E-06 | *** |
| Degree of freedom II | β41 | -138,856 | 18,508 | -7.502 | 6.39E-14 | *** |
| Degree of freedom III | β42 | -148,328 | 20,181 | -7.350 | 2.02E-13 | *** |
| Degree of freedom IV | β43 | -174,966 | 20,916 | -8.365 | <2e-16 | *** |
| Transit classification Escort | β51 | 8,840 | 16,929 | 0.522 | 0.60153 |  |
| Transit classification Independent | β52 | -17,969 | 18,403 | -0.976 | 0.32885 |  |
| Age | β6 | -1,470 | 277 | -5.304 | 1.14E-07 | *** |
| Living in secondary medical area | β7 | -37,592 | 6,162 | -6.101 | 1.06E-09 | *** |
| MDC02 | β802 | -698,324 | 16,093 | -43.393 | <2e-16 | *** |
| MDC03 | β803 | -414,345 | 19,075 | -21.722 | <2e-16 | *** |
| MDC04 | β804 | -241,134 | 15,195 | -15.869 | <2e-16 | *** |
| MDC05 | β805 | 284,526 | 15,622 | 18.213 | <2e-16 | *** |
| MDC06 | β806 | -433,938 | 13,935 | -31.140 | <2e-16 | *** |
| MDC07 | β807 | -257,613 | 16,619 | -15.501 | <2e-16 | *** |
| MDC08 | β808 | -417,814 | 23,622 | -17.687 | <2e-16 | *** |
| MDC09 | β809 | -517,055 | 29,248 | -17.678 | <2e-16 | *** |
| MDC10 | β810 | -411,092 | 19,117 | -21.505 | <2e-16 | *** |
| MDC11 | β811 | -459,938 | 18,439 | -24.943 | <2e-16 | *** |
| MDC12 | β812 | -418,396 | 16,460 | -25.420 | <2e-16 | *** |
| MDC13 | β813 | -37,464 | 20,840 | -1.798 | 0.07223 | . |
| MDC14 | β814 | -414,220 | 61,306 | -6.757 | 1.43E-11 | *** |
| MDC15 | β815 | -359,636 | 60,477 | -0.998 | 0.31845 |  |
| MDC16 | β816 | -380,782 | 24,656 | -15.444 | <2e-16 | *** |
| MDC17 | β817 | -158,040 | 33,813 | -4.674 | 2.96E-06 | *** |
| MDC18 | β818 | -141,131 | 26,442 | -5.337 | 9.48E-08 | *** |
| With surgery | β9 | 349,099 | 6,650 | 52.495 | <2e-16 | *** |

*: p<0.05, **: p<0.01, ***: p<0.001

MDC, Major Diagnostic Categories
