# Supplementary material for: Impact of cognitive decline on medical outcomes and nursing workload: A retrospective cohort study
Source: PLoS One. 2023 Nov 22;18(11):e0293755. doi: 10.1371/journal.pone.0293755 (PMC10664958; doi:10.1371/journal.pone.0293755)
Supplement: S4 Table — (DOCX) [file pone.0293755.s004.docx]

S4 Table. Results of Regression Analysis Using the Frequency of Nursing Care Provided per Day as a Dependent Variable.

| Explanatory variable | Parameter | Estimate | Standard error | t value | Pr (>\|t\|) |  |
| --- | --- | --- | --- | --- | --- | --- |
| (Intercept) | β0 | 134.16509 | 1.50100 | 89.384 | <2e-16 | *** |
| A: Dementia disease name | β1 | 8.66048 | 1.78651 | 4.848 | 1.25E-06 | *** |
| B: Dementia treatment | β2 | -4.74309 | 1.91984 | -2.471 | 0.01349 | * |
| C: Assessment by nurse | β3 | 13.96522 | 1.20931 | 11.548 | <2e-16 | *** |
| Degree of freedom II | β41 | -21.25777 | 1.13367 | -18.751 | <2e-16 | *** |
| Degree of freedom III | β42 | -36.57384 | 1.23613 | -29.587 | <2e-16 | *** |
| Degree of freedom IV | β43 | -41.24335 | 1.28115 | -32.192 | <2e-16 | *** |
| Transit classification Escort | β51 | -44.47572 | 1.03694 | -42.891 | <2e-16 | *** |
| Transit classification Independent | β52 | -47.63916 | 1.12721 | -42.263 | <2e-16 | *** |
| Age | β6 | 0.18144 | 0.01697 | 10.689 | <2e-16 | *** |
| Living in secondary medical area | β7 | 3.60053 | 0.37743 | 9.540 | <2e-16 | *** |
| MDC02 | β802 | -35.05319 | 0.98573 | -35.561 | <2e-16 | *** |
| MDC03 | β803 | -16.02339 | 1.16839 | -13.714 | <2e-16 | *** |
| MDC04 | β804 | -10.43083 | 0.93073 | -11.207 | <2e-16 | *** |
| MDC05 | β805 | -11.44015 | 0.95688 | -11.956 | <2e-16 | *** |
| MDC06 | β806 | -4.80723 | 0.85356 | -5.632 | 1.79E-08 | *** |
| MDC07 | β807 | -19.57937 | 1.01793 | -19.234 | <2e-16 | *** |
| MDC08 | β808 | -30.44041 | 1.44692 | -21.038 | <2e-16 | *** |
| MDC09 | β809 | -13.77752 | 1.79153 | -7.690 | 1.50E-14 | *** |
| MDC10 | β810 | -10.31376 | 1.17094 | -8.808 | <2e-16 | *** |
| MDC11 | β811 | -7.79244 | 1.12946 | -6.899 | 5.30E-12 | *** |
| MDC12 | β812 | -10.30276 | 1.00819 | -10.219 | <2e-16 | *** |
| MDC13 | β813 | -12.37790 | 1.27648 | -9.697 | <2e-16 | *** |
| MDC14 | β814 | -9.84791 | 3.75517 | -2.622 | 0.00873 | ** |
| MDC15 | β815 | -76.80376 | 22.08019 | -3.478 | 0.00051 | *** |
| MDC16 | β816 | -13.84654 | 1.51027 | -9.168 | <2e-16 | *** |
| MDC17 | β817 | -10.06364 | 2.07114 | -4.859 | 1.18E-06 | *** |
| MDC18 | β818 | -13.91718 | 1.61963 | -8.593 | <2e-16 | *** |
| With surgery | β9 | 22.17032 | 0.40734 | 54.427 | <2e-16 | *** |

*: p<0.05, **: p<0.01, ***: p<0.001

MDC, Major Diagnostic Categories
