# Supplementary material for: Impact of cognitive decline on medical outcomes and nursing workload: A retrospective cohort study
Source: PLoS One. 2023 Nov 22;18(11):e0293755. doi: 10.1371/journal.pone.0293755 (PMC10664958; doi:10.1371/journal.pone.0293755)
Supplement: S5 Table — (DOCX) [file pone.0293755.s005.docx]

S5 Table. Results of Regression Analysis Using the Number of Characters in Nursing Records per Day as a Dependent Variable.

| Explanatory variable | Parameter | Estimate | Standard error | t value | Pr (>\|t\|) |  |
| --- | --- | --- | --- | --- | --- | --- |
| (Intercept) | β0 | 509.12 | 7.17 | 70.962 | <2e-16 | *** |
| A: Dementia disease name | β1 | 83.09 | 8.53 | 9.738 | <2e-16 | *** |
| B: Dementia treatment | β2 | 8.17 | 9.18 | 0.890 | 0.37341 |  |
| C: Assessment by nurse | β3 | 112.79 | 5.79 | 19.497 | <2e-16 | *** |
| Degree of freedom II | β41 | -64.15 | 5.42 | -11.837 | <2e-16 | *** |
| Degree of freedom III | β42 | -142.84 | 5.91 | -24.172 | <2e-16 | *** |
| Degree of freedom IV | β43 | -166.27 | 6.13 | -27.146 | <2e-16 | *** |
| Transit classification Escort | β51 | -126.50 | 4.96 | -25.518 | <2e-16 | *** |
| Transit classification Independent | β52 | -164.39 | 5.39 | -30.505 | <2e-16 | *** |
| Age | β6 | 0.85 | 0.08 | 10.416 | <2e-16 | *** |
| Living in secondary medical area | β7 | 14.06 | 1.81 | 7.787 | 6.99E-15 | *** |
| MDC02 | β802 | -178.03 | 4.72 | -37.755 | <2e-16 | *** |
| MDC03 | β803 | -1.66 | 5.59 | -0.297 | 0.76673 |  |
| MDC04 | β804 | 15.16 | 4.45 | 3.405 | 0.00066 | *** |
| MDC05 | β805 | 22.82 | 4.58 | 4.987 | 6.15E-07 | *** |
| MDC06 | β806 | 23.38 | 4.08 | 5.726 | 1.03E-08 | *** |
| MDC07 | β807 | -73.62 | 4.87 | -15.119 | <2e-16 | *** |
| MDC08 | β808 | -10.29 | 6.92 | -1.487 | 0.13700 |  |
| MDC09 | β809 | 21.62 | 8.57 | 2.523 | 0.01166 | * |
| MDC10 | β810 | -38.26 | 5.60 | -6.829 | 8.68E-12 | *** |
| MDC11 | β811 | -9.28 | 5.40 | -1.719 | 0.08567 | . |
| MDC12 | β812 | -68.39 | 4.82 | -14.185 | <2e-16 | *** |
| MDC13 | β813 | -17.46 | 6.11 | -2.859 | 0.00425 | ** |
| MDC14 | β814 | 28.92 | 17.97 | 1.609 | 0.10754 |  |
| MDC15 | β815 | -110.75 | 105.65 | -1.048 | 0.29455 |  |
| MDC16 | β816 | -29.22 | 7.22 | -4.047 | 5.19E-05 | *** |
| MDC17 | β817 | 91.30 | 9.88 | 9.242 | <2e-16 | *** |
| MDC18 | β818 | -27.00 | 7.74 | -3.486 | 0.00049 | *** |
| With surgery | β9 | 99.76 | 1.95 | 51.203 | <2e-16 | *** |

*: p<0.05, **: p<0.01, ***: p<0.001

MDC, Major Diagnostic Categories
